# Strategies to overcome barriers to the statistical representation of femicide data-a technical note

**DOI:** 10.1007/s00414-025-03419-z

**Published:** 2025-01-27

**Authors:** Reena Sarkar, Richard Bassed, Joan Ozanne-Smith

**Affiliations:** 1 Department of Forensic Medicine, Monash University, Victoria, Australia; 2 Victorian Institute of Forensic Medicine, Victoria, Australia

**Keywords:** Femicide, Data system, Statistical representation, Gender motive

## Abstract

Mortality data systems are upstream determinants of health, providing critical information on causes of death and population health trends and influencing health outcomes by shaping policies, research, and resource allocation. Moreover, the gender-related deaths of women and girls are significantly underrepresented or underrecognized in mortality data across many countries. This paper seeks to identify potential barriers and facilitators to improving the representation of femicide data. The primary barriers affecting data representation of femicide are related to definitions, data collection, coding, comparability, access, and systemic challenges. Key recommendations include establishing a nationwide consensus on the definition of femicide, updating training modules for medicolegal professionals, improving pathology reporting processes, ensuring quality assurance in documentation, refining coding practices, developing new analytic methods, and providing deidentified access to cases still under investigation.

## Introduction

Measuring violence and its gender dimensions is essential for monitoring and reducing femicide, the gender-related killing of women and girls [1, 2], recognized by the United Nations (UN) Sustainable Development Goal (SDG) indicators 5.2.1 and 5.2.2 [3]. Mortality data systems are crucial for informing public health policies, shaping resource allocation, and advancing health equity [4]. In 2021, an estimated 81,100 women and girls were intentionally killed, but 45% lacked contextual information to classify them as gender-related killings, resulting in approximately 36,100 undetermined female homicides [5]. This paper discusses barriers to femicide data representation, including the lack of a unified definition, incomplete data collection, coding inconsistencies, limited dataset comparability, restricted data access, and systemic biases (see Table 1). The recommended data-driven strategies in femicide reporting have the potential to significantly enhance prevention strategies and policy.

**Table 1:** Barriers to femicide reporting and strategies for overcoming these limitations

Barriers	Recommendations
Definitional consensus	• Multiagency consultation for standardization • Embed femicide in the legal framework
Data collection	• Updated stakeholder training on femicide specific topics • Interagency information sharing • Integrating technology in data collection systems • World Health Organisation-compliant autopsy reporting
Coding	• Embrace updated coding systems
Comparability	• Multiagency cooperation • Link to services’ datasets
Access and timeliness	• Access to cases still under investigation • Assigning provisional victim-perpetrator relationship • Considering alternative information sources
Systemic bias	• Enhanced dataset for both the deceased and perpetrators • Statistical techniques • Conducting local studies

A number of recommendations follow in response to the barriers to reporting of femicide described above. The primary recommendations are for the standardization of definitions and data collections for, at least, an agreed core dataset of femicide cases to ensure comparability over time and between countries or regions. Additional categories to meet local definitions and legal requirements could be compartmentalized in each data system, again, using standardized definitions and datasets where possible. The specific role of research at each of these steps would be to evaluate the effectiveness of the intervention by means of methods including results for a demonstration-project or by means of a qualitative study involving end users (for instance, an online survey of national experts) [6].

The order in which other recommendations are implemented will depend very much on local needs, circumstances and opportunities. Besides unified definitions, streamlined documentation, embedding the data system in legislation, and gender-sensitive training are integral to femicide reporting.

## Methods

R.S’s doctoral thesis conducted from 2017 to 2021 (degree conferred 2021) contributed to the majority of observations of this viewpoint [7]. Familial, intimate partner and child homicide in Victoria, Australia were studied through combined injury, epidemiologic, and data-system perspectives. A literature search with the search terms “killings of women and girls”, “femicide”, “selective feticide” “data barriers”, “data” was used for supplementation with the most recent updates.

For the literature updates, we searched the databases for medicine, nursing and health sciences (including analysis and policy, criminology), general and grey literature. Examples of the included databases were: Proquest, Informit, PsycInfo, EBSCOhost, Google Scholar and the News. National and international organizational websites such as the Institutes for Criminology, United Nations and World Health Organization were scanned for relevant resources. Eligible study designs were primary studies, organizational reports, and English language literature reviews spanning the period 2017–2024.

## Definitional consensus

Reaching consensus on femicide definitions and indicators is difficult due to differing sociocultural and political contexts [8]. Definitions vary by region, such as the persistence of female infanticide in some areas [9, 10]. Table 2 demonstrates some of the leading regional and organizational definitions and situational context from the recent literature (2014–2025). The Table illustrates definitional variations through examples/indicators providing insight into the underlying issues. Indicators are defined as combinations of data that form the basis for measurements and comparison of femicide over time and across regions [6]. The specifications relate to case characteristics such as the circumstances, victim-perpetrator relationship criteria, urban/rural location, and mechanism of inflicting fatal injuries.

**Table 2:** Femicide definitions and examples between 2014 to 2025

Organization/Region	Year	Definition	Examples/Indicators
The **Latin American** Model Protocol [11] for the Investigation of Gender-Related Killings of Women (Femicide/Feminicide)-UN Women, UN Human Rights Regional Office for Central America	2014	The murder of women because they are women, whether it is committed within the family, a domestic partnership, or any other interpersonal relationship, or by anyone in the community, or whether it is perpetrated or tolerated by the state or its agents	Should be systematically applied to all cases involving the violent death of a woman regardless of suspicion of foul play
The **Inter-American Convention** to Prevent, Punish and Eradicate Violence Against Women [12] (Inter-American Commission of Women. Follow-up Mechanism to the Belém do Pará Convention)	2018	Any gender-based action or conduct that causes the death, injury, or physical, sexual, or psychological suffering of a woman in the public or private sphere that is motivated by or sustains the historically unequal power relations between men and women and places women in situations of subordination, that constitutes a violation of their human rights that it totally or partially limits the recognition, enjoyment, and exercise of such rights	1. Omission of therapeutic abortion, 2. Femicide suicide 3. by induction or aid, obstruction of access to justice, 4. Other aggravating factors when committed against women (aggressor is an agent of the State, victim is an elderly woman, or imprisoned or culturally diverse, migrant, refugees, pregnant, disabled, socioeconomically weak, or in a situation of armed conflict, political violence, sexual exploitations, labour exploitation, human trafficking, or natural disasters) 5. Inflict humiliating or scornful injuries on the corpse of the woman, 6. *Signs of violence* such as hanging, strangulation, suffocation, drowning and immersion and/or injuries caused by sharp objects, substances and fires, or blunt objects 7. Offence is committed in the presence of older and/or younger relatives of the victim or any person under eighteen years old
Cecchi et al.[8] (Italy, the United Kingdom, Japan)	2022	Femicide should be defined as a murder perpetrated because of a failure to recognize the victim’s right to self-determination	Honour crime, fatal intimate partner violence, other domestic violence (perpetrated by family members), femicide in the context of social inequality, armed conflicts, and migration, and femicide due to patriarchal culture prevalent in *rural* areas
UNODC, UN Women [1] (Statistical framework)	2022	Femicide/feminicide should include killings with the following characteristics: i. the killing of a woman by another person (objective criterion); ii. the intent of the perpetrator to kill or seriously injure the victim (subjective criterion); iii. the unlawfulness of the killing (legal criterion); iv. the gender-related motivation of the killing “Gender-related motivation”, the term used to lay the foundation of the statistical definition of gender-related killings of women and girls (femicide/feminicide), refers to the root causes – such as stereotyped gender roles, discrimination towards women and girls, inequality and unequal power relations between women and men in society – that characterize the specific context in which such killings take place	1. Homicides of women by intimate partners (current/former/dating) 2. Killings by family members (male/female) other than intimate partners (honour killings, dowry killings) 3. The perpetrator occupies a position of authority or care over the female victim (victim’s doctor, nurse, teacher, police officer, public official or clergy) 4. Killings of women rooted in gender-related motivations encountered in the context where there was no previous relationship, or in cases where the perpetrator may remain unknown to responsible national authorities Eight indicative characteristics: 1. Previous record of physical, sexual, or psychological violence/harassment perpetrated by the author of the killing; 2. Was a victim of forms of illegal exploitation, (trafficking in persons, forced labour or slavery); 3. Homicide victim was abducted or illegally deprived of her liberty; 4. The victim was working in the sex industry; 5. Sexual violence against the victim was committed before and/or after the killing; 6. The killing was accompanied by mutilation of the body of the victim; 7. The body of the victim was disposed of in a public space; 8. The killing of the woman or girl constituted a gender-based hate crime
UNODC and UN Women [13] (Global estimates)	2023	Femicide (or feminicide, as it is referred to in some contexts) is defined as an intentional killing with a gender-related motivation. It is different from homicide, where the motivation may not be gender related	1. Killing in the context of hate crime 2. Victim working in the sex industry 3. Sexual violence on victim before/after the killing 4. Mutilation of victim’s body 5. Body of victim disposed of in public space
**Canadian** Femicide Observatory for Justice and Accountability [14]	2024	Sex/gender-related killings of women and girls	1. Women and girls killed by intimate partners 2. Women and girls killed by family members 3. Previous record of harassment/violence 4. Illegal deprivation of her liberty 5. Use of force and/or mutilation 6. Body disposed of in a public space 7. Sexual violence was committed before 8. Victim was working in the sex industry 9. Hate crime motivated by bias against women/girls 10. Victim of forms of illegal exploitation (slavery, human trafficking) 11. Killed when they attempt to exert independence over their life or sexuality 12. Killed after being a stalked by their killer for any period of time 13. Killed after being cyberstalked by their killer for any period of time
**European** Institute for Gender Equality [15]	2025	General definition: Killing of women and girls because of their gender Statistical definition: The killing of a woman by an intimate partner and the death of a woman as a result of a practice that is harmful to women. Intimate partner is understood as a former or current spouse or partner, whether or not the perpetrator shares or has shared the same residence with the victim	Five types of contexts are mapped for the European region [16]: political, societal, criminal, sexual and interpersonal. Examples are: 1. Intentional killings of women by an intimate partner and/or family member(s) a. Intentional killing of a woman by an intimate partner (including current or former partners, living in the same household or not) b. Intentional killing of a woman by family member(s) i. Honour killing ii. Dowry-related killing c. Other intentional killing of a woman by family member(s) 2. Other types of intentional killings a. Killing of a woman by non-family member(s) involving sexualised violence b. Sexual-exploitation-related killing of a woman (with the exception of trafficking-related killing) c. Trafficking-related killing of a woman d. Killing of a woman in the context of a continuum of violence in particular settings (including the killing of a woman by carers or persons in authority, killing of political activists, hate killing) i. From an authority/political group ii. In a care relationship e. Killing of a woman older than 65 by non-family members f. Other types of intentional killing of a woman not listed above 1. Targeted killing of women and girls in the context of armed conflict; 2. Killing of women and girls because of their sexual orientation and gender identity; 3. The killing of aboriginal and indigenous women and girls because of their gender; 4. Female infanticide and gender-based sex selection foeticide; 5. Accusations of witchcraft; and 6. Other femicides connected with gangs, organized crime, drug dealers, human trafficking and the proliferation of small arms 3. Unintentional killings of women a. Death of a woman resulting from intimate partner violence b. Female genital mutilation-related death c. Other types of unintentional killing of a woman not included above

*UN* United Nations, *UNODC* United Nations Office on Drugs and Crime

Challenges include inconsistent data documentation, misclassified or missing information, and a lack of agreement on gender indicators [2, 17]. The UN’s SDGs 5.2.1 and 5.2.2 provide guidance on indicators including victim rates and violence types [3] but omit issues such as selective feticide, infanticide, and violence against LGBTQ + women. The Council of Europe recommends five key indicators: victim and perpetrator sex, the sexual nature of assault, relationships, and gender motivation [2]. Multiregional studies between 2021–2022, across seven European States (Germany, Spain, France, Italy, Lithuania, Finland and Sweden) substantiated these indicators. Methods employed were: questionnaire completed by National researchers on whether statistical data was available on the variables pertaining to the indicators, and if that data could be cross-referenced and disaggregated (e.g., by sex, or by disability status) [18].

Since 2015, data initiatives in Latin America [19], South Africa [20], and Spain [21], have aimed to standardize femicide definitions through participatory action research. In Latin America, studies in countries such as Argentina, Uruguay, and Panama identified barriers and best practices, resulting in a database of 44 variables to analyze femicide in Panama. An example of participatory action research was seen in the South African model that developed a standardized definition using a socioecological framework after a six-month consultation, reclassifying female child sexual abuse as sexual femicide. In Spain, collaboration between police, academics, and courts improved the use of femicide risk assessment tools and introduced targeted interventions for perpetrators.

A modified Delphi study establishing a pilot violence/injury observatory in Cape Town, South Africa in 2017 [22] illustrates a further research methodology. The Delphi study is practical in problematic areas where knowledge is uncertain and incomplete [23]. The researchers developed consensus by two rounds for 14 (of original 21) violence-related indicators and 12 (of 13) violence-related datasets that were deemed as context appropriate by the participants. The participants belonged to a variety of fields such as government and community organizations, emergency medicine, research, police and universities representing regional stake holding in violence reduction.

### Recommendations

#### Multiagency consultation for standardization

Multiagency consultation is the recommended method for developing definitional consensus and guidance on gender indicators at a regional level. A consensus should be drawn on key aspects such as comparability with other definitions, on the identification of gender motives, and femicide intersections with harmful social practices (e.g., honor killings, dowry deaths).

#### Embed femicide in the legal framework

Such multiagency regional consensus should be adapted in national law, making it more visible and legally distinct from other homicides. Latin American countries that have actively incorporated femicide into their legal framework, can potentially serve as precedents for the implementation of femicide laws in other nations. Similar recommendation for criminal regulation of femicide was concluded after a national online survey of participating European Union countries and the United Kingdom [6].

## Data collection

Administrative data are not well-structured for femicide research [24], as gender-disaggregated data are inconsistently collected across institutions and often lack details on the victim-perpetrator relationship or motive. Additionally, data on sexual orientation and gender identity are frequently omitted, leaving gender-diverse and non-heterosexual populations underrepresented [25]. Sensitive circumstances surrounding gender-based violence may also prevent the recording of gender orientation to avoid further trauma. Biases related to race, ethnicity, and economic status contribute to the underreporting and misclassification of femicide. Issues in death registration systems, such as low-quality cause of death data [26], further obscure femicide distributions. In India, the availability of registered and medically certified causes of death slightly increased from 17.1% to 20.6% between 2010 and 2019. However, certain regions such as Goa, Manipur, and Delhi exhibited significantly greater availability, ranging from 61.7% to 100% [27].

### Recommendations

#### Updated stakeholder training on femicide specific topics

Data collection barriers can be minimized by improving the consistency of investigative data and cause of death reporting. The sensitization and training of police officers, judges, hospital and health workers, medical coders, mortuary staff, forensic doctors and researchers about gender-based violence is needed to make gender visible in documentation [10, 28–30].

#### Interagency information sharing and integrating technology in collection systems

Understanding the patterns of abuse, and information sharing between stakeholders (e.g., with forensic services) and integrating technology in reporting can help police efficiently record the underlying gender motivations for certain homicides, allowing these to be classified correctly as femicides. This approach would also encourage victim reporting due to a sense of trust and safety whilst disclosing abuse.

#### World Health Organization-compliant autopsy reporting

Standardized forensic pathology *autopsy* reporting should accord with the World Health Organization (WHO) prescribed medical cause of death certification format and the International Classification of Diseases (ICD) 2016 manual. Regular assessment of the quality and usability of death certification and timely availability of state/provincial/country level causes of death registries will ensure that administrative crime data, compiled according to national legislation [31], are harmonized.

## Coding

The current ICD-10 system employs codes for gender-related issues, but medical coders often face ambiguity in applying these codes due to inconsistent case documentation [32–34]. The ICD-10 codes are broad and do not effectively capture motives or contexts. Coders may lack training in ambiguous cases and often interpret documentation subjectively. They rely on varied sources, such as medical records and police reports, which may lack consistent detail and terminology. As a result, if gender-based violence is not clearly indicated, coders may default to broader classifications. In contrast, ICD-11 codes reflect modern understanding of sexual health and gender identity by replacing ICD10 diagnostic categories like “transsexualism” with “gender incongruence of adolescence and adulthood” [35]. Also, gender incongruence has been moved out of the “Mental and behavioural disorders” chapter and into the new “Conditions related to sexual health” chapter.

### Recommendations

#### Embrace updated coding systems

The International Classification of Crime for Statistical Purposes (ICCS) and the ICD11 are effective coding systems that possess the capacity to cater to the complex data needs of femicide. The ICCS, developed by the UN Economic Commission for Europe and the UN Office on Drugs and Crime, classifies intentional homicide accounting for obscure gender motives, such as in positions of authority (e.g. work/carer relationships) [31]. The Eurostat, based on the crime statistics of all European Union member States and Turkey, has embraced the ICCS system to classify intentional violence data (including homicide and sexual violence) [6].

Compared with earlier versions, ICD11 offers enhanced flexibility for detailed coding [36]. Its Digital Open Rule Integrated Cause of Death Selection (DORIS) software automates the selection of the underlying cause of death from death certificates [37]. DORIS operates by an autocomplete search feature where the search engine predicts the input query for administrative data, cause of death 1a-d, manner of death, place of occurrence, and provides suggestions for replacing the corresponding selections by ICD11 codes. The output section also includes capacity to accommodate multiple underlying causes of death unlike ICD10. For example: a 47-year-old female with a history of psychological abuse, murdered by her partner at home by the mechanisms of blunt force and ligature strangulation would be captured as a string of codes in an output CSV-file. Using post coordination function, the output information would be able to capture details such as sex, age, place of death, immediate cause of death (NA02.8, PE6Z&XE11D) including object use (XE11D), underlying causes (PE40/NA02.8) and contributing condition (relationship distress, QE51.0). While the software is freely accessible, its full potential will be realized when the ICD11 is integrated into health service systems including mortality statistics. An Emergency Department (ED) based study examining the sensitivity and specificity of ICD10 coding in clinical surveillance of intimate partner violence (IPV) reported a sensitivity of 33% (95% confidence interval/CI 26.1% to 41.2%) in 55 of 165 eligible cases for IPV-related care within one health care system between February 1, 2021, and June 14, 2022 [38]. The ICD10 codes displayed moderate specificity only (85.7%, 95% CI 83.3% to 87.8%).

## Comparability

The lack of coordination among datasets from various administrative units, such as vital statistics, police, courts, hospitals, and population surveys, poses significant challenges in collecting accurate data on femicides. Fragmented data across agencies obstructs understanding of the true cause distribution and resource allocation. Limited data sharing between sectors such as forensic services and police results in disjointed and non-comparable information. Femicides may be classified as accidents or suicides in different datasets due to this lack of coordination. Additionally, disparities in resource allocation for data collection exist across regions; for example, India’s long-standing civil registration system remains underutilized due to inconsistent geo-social efforts and inadequate multi-state coordination [26]. An analysis of pan-European (European Union and United Kingdom) data-collection systems reported a need for “substantial investment” for quality data collection [16, 18].

### Recommendations

#### Multiagency cooperation

Multiagency cooperation can significantly improve the quality of femicide data. In Cali, Colombia, police, the attorney-general’s office, and the Institute of Legal Medicine meet weekly to consolidate data on violent deaths [39]. Similarly, the Pasto Crime Observatory expanded its dataset to include nonfatal injuries, child abuse, and gender-based violence, sharing reports with local authorities. Quality assurance involves experts such as epidemiologists and psychologists [40]. The EIGE research strongly recommends inter-institution coordination as a productive method to standardize measurement. Their research showed that a dedicated body that coordinates the collection of data exists in nine Member States (Ireland, Spain, France, Croatia, Italy, Luxembourg, Malta, Austria, Portugal) [16]. In Spain, femicide data is collected through the Spanish Government Office against Gender-

based Violence, under the Ministry of Equality synergizing inputs from multiple Cabinets (Ministry of Justice, the Ministry of Health, the Ministry of the Interior, and the Spanish Bar Association) [6].

#### Link to services’ datasets

Linking or matching homicide data with administrative data from services (e.g., hotlines, shelters) can help track survivors and analyze help-seeking behaviors [29]. In Mozambique, the UN and Ministry of Interior launched *InfoViolencia*, a platform for managing gender-based violence cases, which may inform future femicide prevention [41].

## Data access and timeliness

Access to homicide data may be fully or partially restricted [42, 43] due to the complexities associated with death investigation systems. As criminal investigations or database coding may be ongoing, some data repositories restrict access to such cases, leading to data being unavailable for statistical reports and research. Access issues may also result from unusual delays in formal release of causes of death [26].

### Recommendations

#### Access to cases still under investigation

Access to cases labelled ‘still enquiring’ or ‘under-investigation’ can help accurately estimate femicide numbers. This recommendation may need to be balanced with consideration of the time needed to conduct the coronial investigation in an efficient and thorough manner [44]. Datasets offering deidentified data for open cases may be more fit for the purpose of reporting long-term trends on femicide [45]. For statistical purposes such as computing the annual change in intimate partner femicide, the impact exerted by a potential change to case numbers is likely minor. In Studdert and Cordner’s study of deaths from all causes reported to Coroners in Australia [46], between 2000 and 2007, the authors noted a difference in only 5.2% (6222/120,452) of cases having presumptive intent and manner of death changed due to a completed coronial investigation. For confirmed homicides, the Australia-wide net reduction in the number of cases was minimal (6.1 annually).

#### Assigning provisional victim-perpetrator relationship

Several homicide datasets include provisional victim-perpetrator relationships for these cases enabling their inclusion in problem size estimates [45].

#### Considering alternative information sources

In countries with limited surveillance capacities, alternative models such as media-based systems can be effective, as seen in Israel, Turkey, in the Minnesota Femicide Report, United States of America [6], and Mexico’s National Citizen Observatory where police data are lacking [47, 48], providing insights into homicide between lesbian, gay, bisexual and/or transgender intimate partners [6]. Open-source homicide data may be a viable alternative to official sources, although they share limitations such as potential missing values, being labour intensive, and having reliability issues similar to conventional data sources [43].

## Systemic bias

Regardless of the countries’ development indices, an underfunded and under-optimized medicolegal death investigation system is one of the major contributors to systemic bias regarding femicide. Gender-oblivious beliefs within the legal system may prevent the detection of femicide on the premise of *insufficient* evidence to prove gender motivation for homicide, insidiously augmenting data bias [17]. A South African study suggested that violent crime figures are often underreported to pose an illusion of law and order [49].

The use of data management operating systems may also introduce bias into homicide data. A 2021 Australian study highlighted inconsistencies in reporting family violence homicide between data systems. Using Joinpoints regression, the study compared family violence homicide rate trends from three data sources and found, depending on the data system, an uptrend or downtrend [45].

### Recommendations

#### Enhanced dataset for both the deceased and perpetrators

We recommend a multi-dataset, observatory study design to address incomplete data and identify biases. A meta-analysis on implementation mapping of injury observatories showed a reduction in homicide and assault rates in high-violence areas [50]. Most injury surveillance systems follow the WHO’s minimum dataset recommendations based on the International Classification of External Causes of Injury, with some using geospatial mapping to identify at-risk populations. Security and data visualization tools ranked highly for prototype systems [51]. An enhanced homicide register, which captures details about victims and perpetrators, is ideal for determining gender motivation and guiding public policy [52]. Such observatories or homicide monitoring systems exist across world regions such as Latin America, Australia, European Union, United States and the United Kingdom [6].

#### Statistical techniques

Disaggregating data by factors such as age, sex, ethnicity, substance abuse status, mental health issues and socioeconomic status helps establish motives for femicide, while methods like Joinpoints, Poisson regression, and time series analysis can elucidate temporal trends [39, 45, 53].

#### Conducting local studies

City level [54] or localized studies with smaller datasets, often used in suicide and homicide research [7, 55], are valuable for monitoring complex femicide cases. For example, case management systems of state forensic medical services have local data such as forensic imaging and computed tomography. To mitigate data bias from missing gender orientation information, qualitative studies (e.g., anonymized surveys) can supplement quantitative data, revealing experiences of gender-based violence. Thematic analyses, typically used in intimate partner violence studies [56, 57], could also be applied to femicide.

## Challenges

Implementing femicide prevention strategies may be beset with challenges such as cultural resistance or resource and technology needs. Low-resource areas need substantial investments to establish basic infrastructure, while high-resource regions incur expenses for upscaling services. The benefits such as reduced violence, increased social stability, improved public trust, and potential global leadership outweigh the costs in both settings.

## Limitations

Recommendations concerning other operability measures such as the efficacy of special prosecuting courts, special forensic units and female judges [58] are outside the scope of this paper. There are several legislative challenges involved in specifically criminalizing femicide, however the paper is limited only to data-related issues. Many world regions are demonstrating initiatives in femicide response; however, it was not possible to translate all femicide-specific guidelines published in region-specific languages.

## Conclusion

This article highlights barriers to accurately representing femicide in communities and proposes solutions. Effective documentation and unified definitions are essential first steps in recognizing femicides globally. Achieving gender visibility in case recording is complicated by various issues in medicolegal death investigations and regional factors. Countries should implement legislated, gender-sensitive data collection systems for femicide. Sustainability requires political will, resource allocation for injury surveillance, and ongoing gender-sensitive training to identify femicide trends. Future research should examine the influence of cultural, religious, and social norms on the prevalence of femicide and the role of technology in predicting and preventing it.

## Data Availability

Not applicable.
